# IDAC-Dose 2.1, an internal dosimetry program for diagnostic nuclear medicine based on the ICRP adult reference voxel phantoms

**DOI:** 10.1186/s13550-017-0339-3

**Published:** 2017-11-03

**Authors:** Martin Andersson, Lennart Johansson, Keith Eckerman, Sören Mattsson

**Affiliations:** 1 Medical Radiation Physics, Department of Translational Medicine, Malmö, Lund University, Skåne University Hospital, SE-205 02 Malmö, Sweden; 2 Radiation Physics, Department of Radiation Sciences, Umeå University, SE-901 87 Umeå, Sweden; 3 Center for Radiation Protection Knowledge, Oak Ridge National Laboratory, Oak Ridge, TN USA

**Keywords:** Internal dosimetry, ICRP, IDAC, DCAL, Effective dose, Radiopharmaceuticals, Diagnostic nuclear medicine

## Abstract

**Background:**

To date, the estimated radiation-absorbed dose to organs and tissues in patients undergoing diagnostic examinations in nuclear medicine is derived via calculations based on models of the human body and the biokinetic behaviour of the radiopharmaceutical. An internal dosimetry computer program, IDAC-Dose2.1, was developed based on the International Commission on Radiological Protection (ICRP)-specific absorbed fractions and computational framework of internal dose assessment given for reference adults in ICRP Publication 133. The program uses the radionuclide decay database of ICRP Publication 107 and considers 83 different source regions irradiating 47 target tissues, defining the effective dose as presented in ICRP Publications 60 and 103. The computer program was validated against another ICRP dosimetry program, Dose and Risk Calculation (DCAL), that employs the same computational framework in evaluation of occupational and environmental intakes of radionuclides. IDAC-Dose2.1 has a sub-module for absorbed dose calculations in spherical structures of different volumes and composition; this sub-module is intended for absorbed dose estimates in radiopharmaceutical therapy. For nine specific alpha emitters, the absorbed dose contribution from their decay products is also included in the committed absorbed dose calculations.

**Results:**

The absorbed doses and effective dose of ^131^I-iodide determined by IDAC-Dose2.1 were validated against the dosimetry program DCAL, showing identical results. IDAC-Dose2.1 was used to calculate absorbed doses for intravenously administered ^18^F-FDG and orally administered ^99m^Tc-pertechnetate and ^131^I-iodide, three frequently used radiopharmaceuticals. Using the tissue weighting factors from ICRP Publication 103, the effective dose per administered activity was estimated to be 0.016 mSv/MBq for ^18^F-FDG, 0.014 mSv/MBq for ^99m^Tc-pertechnetate, and 16 mSv/MBq for ^131^I-iodide.

**Conclusions:**

The internal dosimetry program IDAC-Dose2.1 was developed and applied to three radiopharmaceuticals for validation against DCAL and to generate improved absorbed dose estimations for diagnostic nuclear medicine using specific absorbed fraction values of the ICRP computational voxel phantoms. The sub-module for absorbed dose calculations in spherical structures 1 mm to 9 cm in diameter and different tissue composition was included to broaden the clinical usefulness of the program. The IDAC-Dose2.1 program is free software for research and available for download at http://www.idac-dose.org.

## Background

In radiological protection, it is important to be able to perform valid calculations of the absorbed dose in organs and tissues of the body for persons exposed to external radiation sources and radiation from internally distributed radionuclides. Computer programs are needed because it is difficult to measure the absorbed dose to organs and tissues. For radionuclides entering the body by inhalation and ingestion in occupational and environmental exposures, the Dose and Risk Calculation (DCAL) [1] program has been used to derive nuclide-specific dose coefficients published by the International Commission on Radiological Protection (ICRP). In addition, “Internal Dose Assessed by Computer” (IDAC-Dose) [2, 3] program has been used by ICRP to tabulate dose coefficients for patients undergoing examinations with radiopharmaceuticals in nuclear medicine (mainly intravenous administration, sometimes orally or via inhalation). Similar programs have been developed by other groups (RADAR [4, 5], etc.). The ICRP Task Group on “Radiation dose to patients in diagnostic nuclear medicine” has issued several publications [6–9] on the absorbed dose to patients from different clinically used diagnostic radiopharmaceuticals using the IDAC-Dose1.0 program. DCAL has been used by the ICRP Task Group on “Dose calculations” to estimate doses to occupationally exposed individuals and members of the public [10–15]. At that time, the absorbed dose calculations using DCAL and IDAC-Dose1.0 were based on the stylised family anatomical phantoms described by Cristy and Eckerman [16] and based on the data given in ICRP Publication 23 for the “Reference Man” [17]. The stylised phantoms were described by linear and quadratic equations and included 24 specific organs. In IDAC-Dose1.0, radionuclide-specific absorbed dose calculations were interpolated from monoenergetic photon-specific absorbed fractions (SAFs) between 10 and 4 MeV, and electrons were assumed to be locally absorbed.

An updated set of reference values for basic anatomical and physiological data was given in ICRP Publication 89 [18] for both male and female subjects of six different ages (newborn, 1, 5, 10, and 15 years, and adult) based on statistics for Western Europeans and North Americans. In developing guidelines to limit potential stochastic effects (mainly based on cancer statistics) from radiation exposure (external and internal), the ICRP defines an idealised person called the “Reference Person” and the dosimetric quantity effective dose [19]. To calculate the effective dose, the equivalent doses to the “Reference Male” and “Reference Female” are averaged. The underlying anatomical and physiological reference values for the two reference individuals are presented in ICRP Publication 89 [18]. Voxel phantoms describing these two adults are given in ICRP Publication 110 [20], and SAF values for monoenergetic photons, electrons, and alpha particles, as well as radionuclide-specific neutron SAF values, are presented in ICRP Publication 133 [21].

The introduction of more realistic anatomical models with more detailed biokinetic models regarding the uptake, distribution, and retention of radiopharmaceuticals creates the possibility of more accurate and representative absorbed dose estimations. One of the main challenges for medical internal radiation dosimetry is to select an appropriate biokinetic model describing the uptake, retention, and clearance of the radiopharmaceutical of interest. For internal absorbed dose calculations for the Reference Person, updating the general biokinetic models includes more realistic representations of the alimentary and respiratory tract models. Updated nuclear decay data are also available in ICRP Publication 107 [22], and performing absorbed dose estimations based on the new, more realistic SAF values [21] will allow more detailed absorbed dose calculations and will provide more accurate absorbed dose estimations for patients examined with radiopharmaceuticals. The aim of this work was to update the IDAC-Dose2.0 [23] created to calculate absorbed dose based on earlier published SAF values [24] for the ICRP adult computational voxel phantoms [20]. The updated version, IDAC-Dose2.1, is based on the SAF values published by the ICRP [21] and implements the computational framework for internal dose assessment already used by ICRP for absorbed dose calculations at occupational and public exposures. If reliable biokinetic data for the substances are available, the program enables calculations of absorbed dose and effective dose up to 1252 radionuclides published in ICRP Publication 107. This update allows users to perform absorbed dose calculations based on biokinetic data generated in different research programs.

A secondary aim of this work was to benchmark results obtained from IDAC-Dose2.1 with those obtained using DCAL under the same reference conditions. Both IDAC-Dose2.1 and the new version of DCAL base their dose calculations on the same ICRP computational framework for internal dose assessment [21]. IDAC-Dose2.1 will be used by the ICRP Task Group on “Radiation dose to patients in diagnostic nuclear medicine” for new absorbed dose calculations for patients in future ICRP publications, whereas the DCAL will be used by the ICRP Task Group on “internal dose coefficients” in their publications on dose estimates for occupational and public intake of radionuclides [25, 26].

### Absorbed dose and effective dose

MIRD pamphlet 21 [27] defines the mean absorbed dose (*D*) to a target region (*r*_*T*_) for a time-independent system as Eq. 1:

1 $$ D\left({r}_T,{T}_D\right)={\sum}_{r_S}\overset{\sim }{A}\left({r}_S,{T}_D\right)S\left({r}_T\leftarrow {r}_S\right)\ \left[ Gy\right] $$

where *Ã*(*r*_*S*_, *T*_*D*_) is the time-integrated or cumulated activity (i.e., the total number of disintegrations in source region *r*_*S*_ over the integration period *T*_*D*_. In IDAC-Dose2.1, this integration period is in hours and *T*_*D*_ is normally set to ∞. *S*(*r*_*T*_ ← *r*_*S*_) is the mean absorbed dose in the target tissue per nuclear transformation in the source region and defined as Eq. 2:

2 $$ S\left({r}_T\leftarrow {r}_S\right)={\sum}_i{\Delta }_i\varPhi \left({r}_T\leftarrow {r}_S,{E}_i\right)\ \left[ Gy/ Bq\right] $$

where *Φ*(*r*_*T*_ ← *r*_*S*_, *E*_*i*_) is the SAF value [21] (i.e., the fraction of the *E*_*i*_ emitted in source region *r*_*S*_ to the target tissue *r*_*T*_ divided by the mass of the target tissue in kilograms) of the *i*-th emitted radiation of the radionuclide and ∆_*i*_ = *E*_*i*_ *Y*_*i*_ (the product of *Y*_*i*_, the yield and *E*_*i*_ [22]) is the mean energy (or part of the energy distribution for β-decay) of the *i*-th nuclear transition of the radionuclide in joules.

The effective dose (*E*) is a radiation protection dose quantity defined by the ICRP as the tissue weighted sum of the equivalent doses in specified tissues and organs of the sex-averaged reference person. ICRP has revised the tissue weighting factors twice and, in the latest version presented in ICRP Publication 103, the effective dose to the Reference Person is calculated as Eq. 3:

3 $$ E={\sum}_T{w}_T{\sum}_R\frac{w_R{D}_R{\left({r}_T,T\right)}_{\mathrm{Ref}.\mathrm{male}}+{w}_R{D}_R{\left({r}_T,T\right)}_{\mathrm{Ref}.\mathrm{female}}}{2}\left[ Sv\right] $$

where *D*_*R*_ *(r*_*T*_ *,T)*_Ref. male_ and *D*_*R*_ *(r*_*T*_ *,T)*_Ref. female_ are the mean absorbed doses to the target region for the Reference Male and Reference Female, respectively, for each radiation type *R*; *w*_*R*_is the radiation weighting factor for radiation type *R*, which in this case is 1 for photons and electrons and 20 for alphas; and *w*_*T*_ is the tissue weighting factor, which is the relative contribution from organ and tissue *T* to the total detriment of stochastic effects caused by ionising radiation.

### Input data for IDAC-Dose2.1

**Fig. 1:**
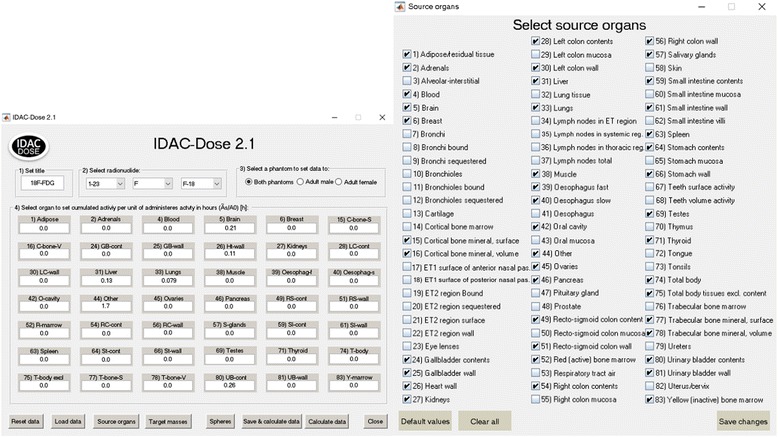
Example of the IDAC-Dose2.1 interface. Left, radionuclide ^18^F has been selected as an example and the cumulated activity per administered activity given in hours from the biokinetic model of ^18^F-FDG in ICRP Publication 128 [9]. Right, the list of all source regions that can be selected in the program

The input data for the absorbed dose calculation part of the program is the total number of disintegrations (or cumulated activity) in a source region (*r*_*S*_) divided by the administered activity (Eq. 4):

4 $$ \frac{\overset{\sim }{A}}{A_0}=\frac{\int_0^{T_D}A\left({r}_S,t\right) dt}{A_0}\left[h\right] $$

where *A*(*r*_*S*_, *t*) is the activity of the radiopharmaceutical in source region *r*_*S*_ at time *t*, and *A*_0_ is the administered activity. *A*(*r*_*S*_, *t*) is integrated up to a time point *T*_*D*_. *A*_0_ should be given in megabecquerel and the integration step must be in hours. In Fig. 1, the cumulated activities per administered activity in various organs and tissues are presented for ^18^F-fluorodeoxyglucose (^18^F-FDG). IDAC-Dose2.1 calculates absorbed doses to all 47 target regions defined in ICRP Publication 133 [21] and two different sets of tissue weighting factors [15, 19] to estimate the effective dose. The results are presented in terms of absorbed dose per administered activity (mGy/MBq) or effective dose per administered activity (mSv/MBq).

### Decay scheme

For dosimetric calculations, ICRP Publication 107 [22] provides information on physical half-lives, decay chains, and the yields and energies of radiation emitted in the nuclear transformations of 1252 radionuclides of 97 elements. This database supersedes the data of ICRP Publication 38 [28] and will be used in future ICRP publications on dose coefficients for the intake of or exposure to radionuclides in the workplace, environment, and nuclear medicine. IDAC-Dose2.1 incorporates the nuclear decay data for all radionuclides and performs absorbed dose calculations for photons, electrons, and alpha particles. IDAC-Dose2.1 also incorporates the Publication 107 beta spectra of 955 radionuclides and calculates radionuclide-specific *S*_beta_(*r*_*T*_ ← *r*_*S*_) values. The *S*_beta_(*r*_*T*_ ← *r*_*S*_) values are generated by integration over the beta particle energy distribution, i.e., the summation in Eq. 2 is replaced with an integral.

### Source regions

The computer program consists of 83 different source regions, 79 of which were addressed in SAF tables of ICRP Publication 133. Three of the source regions not included in the SAF tables are the source regions “Total body”, “Total body excluding content”, and “Other”, which are potential source regions in biokinetic models. Another source region not included is the “Lymph node total” or “Lymphatic nodes”, which is defined by mass weighting of the different lymph nodes in the extrathoracic, thoracic, and systemic regions.

#### Other

For the source region “Other”, previously called Remainder [9], no simulated SAF values are presented in ICRP Publication 133 [21]. The Other gathers all soft tissues not included in the biokinetic model, assuming no enhanced uptake of the radiopharmaceutical, making it specific for each model. The Other source region applies solely to systemic activity (i.e., activity that is present in the blood and subject to distribution throughout body tissues). Other includes regions not identified in the systemic biokinetic models. The content regions of the respiratory and gastrointestinal tract are not part of the Other. The Other region is subject to blood flow and has an assigned mass, excluding surface regions of teeth and bone from the Other. The Other region must be unique (i.e., not a part of a larger region to be included in the Other), excluding the stomach mucosa if stomach wall is included. The SAF values for the source region are calculated as Eq. 5:

5 $$ \varPhi \left({r}_T\leftarrow {r}_{\mathrm{Other}}\right)=\frac{1}{M_{\mathrm{Other}}}{\sum}_{r_{S,O}}{M}_{r_{S,O}}\varPhi \left({r}_T\leftarrow {r}_{S,R}\right)\ \left[k{g}^{-1}\right] $$

where *r*_*S,O*_ is the source region *S* included in the source region Other (*O*), and *M*_Other_ and *M*_*rS,O*_ are the total mass of the Other and each individual source region included in the source region Other.

In IDAC-Dose2.1, the Other can be created from three different source regions, either directly by selecting the source region Other or by using one of the source regions for “Total body” and “Total body excluding body content”, with the computer program subtracting the values of the included source regions in the biokinetic model, generating the corresponding value for the source region of the Other.

#### Blood

The source region “Blood” represents the blood circulating in the body. If the biokinetic model addresses the circulating blood then as a separate source region, the activity contribution of the blood content must be removed from the other source regions (i.e., the cumulated activity in the liver region originated from circulating blood must be removed and only activity in the liver tissue should be accounted for). For biokinetic models that do not include the circulating blood as a source region, the program will automatically account for this by adding parts of the SAF values for the source region Blood (e.g., large vessels and heart content in the source region Other), otherwise this part of the phantoms would not be taken into account in the absorbed dose calculation.

### Target regions

**Fig. 2:**
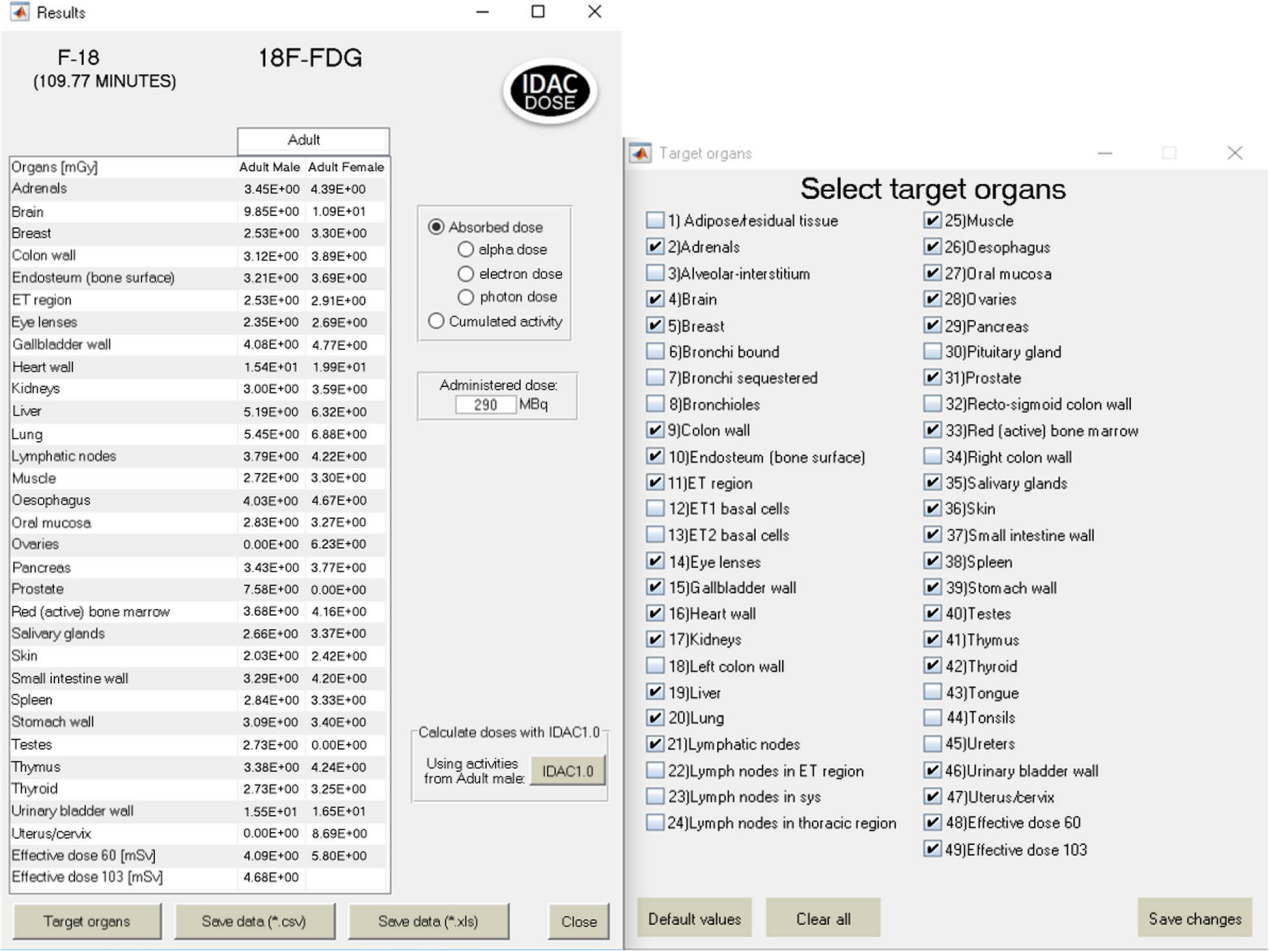
Using IDAC-Dose2.1 to calculate absorbed dose. Left, absorbed dose (in mGy) for the organs and tissues included in the list of tissues with a weighting factor in ICRP Publication 103 and the effective dose as defined in ICRP Publications 60 and 103 (in mSv) together with the absorbed dose to the lens of the eye for the biokinetic model of the intravenous administration of 290 MBq of ^18^F-FDG based on the biokinetic model presented in ICRP Publication 128 [9]. Right, the 47 possible target regions for which absorbed doses are calculated

Forty-seven different target regions are defined in ICRP Publication 133. IDAC-Dose2.1 calculates the absorbed dose to all regions and, if their mass has been altered to adjust for self-irradiation, an additional calculation will be performed. The left part of Fig. 2 shows the absorbed doses for organs and tissues for which tissue weighting factors are given in ICRP Publication 103. The right part of Fig. 2 indicates the possibility to include all 47 target regions and the effective dose as defined in ICRP Publications 60 and 103 in the result list.

### Specific dosimetry

IDAC-Dose2.1 is created for diagnostic nuclear medicine reference dosimetry based on standardised anatomical and biokinetic models for patients. Two tools in the program were prepared for patient-specific dosimetry, which allows adjustments for the activity distribution within an organ or tissue and the ability to estimate absorbed doses to anatomical spherical volumes. These two additions are important for dosimetry in therapy with radiopharmaceuticals.

#### Self-irradiation

When source and target regions are the same, the SAF values can be modified to adjust for variation in the mass of an organ with homogenously distributed activity. For photons, this is performed by adding the factor (*M*_Ref_/*M*_New_)^2/3^, where *M*_Ref_ is the mass of the reference phantoms and *M*_New_ is the new organ mass [29]. For particles, this factor is *M*_Ref_/*M*_New_.

#### Absorbed dose to spherical structures

**Fig. 3:**
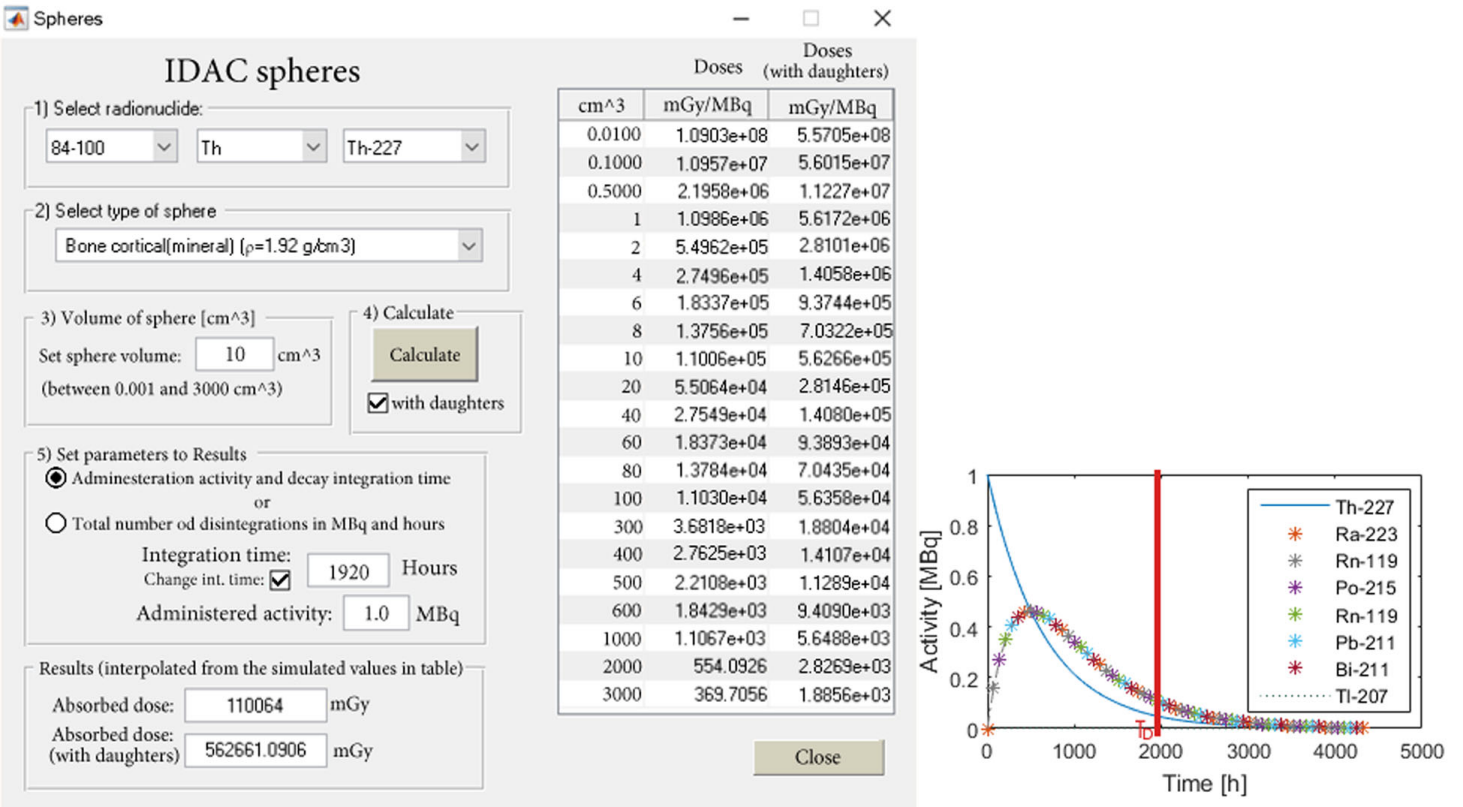
The module to calculate the absorbed dose to various types of spheres. Left, in this example, a “bone cortical” sphere volume of 10 cm^3^ was selected. Right, the activity of ^227^Th (in MBq). The absorbed dose to the sphere was calculated as 110 Gy (563 Gy including the absorbed dose contribution of daughters) for an administration of 1 MBq ^227^Th and an integration period of 80 days (*T*_*D*_ = 1920 h)

A separate sub-module (IDAC Spheres) providing the means to estimate the absorbed dose within spheres of various volumes is included in the program. Spheres have been used to resemble different normal anatomical structures, as well as tumours [30, 31]. The ICRP adult reference computational phantoms consist of 53 different media with different elemental compositions and densities [20] that have been condensed into 26 different types of spheres together with a sphere of water. For each type of sphere, 21 separate monoenergetic photon, electron, and alpha SAF values are generated for volumes ranging from 0.01 to 3000 cm^3^. Data for alpha emitters have been included in the sub-module due to their growing interest in molecular radiotherapy. The SAF values for the spheres are determined assuming a homogeneous distribution within the sphere using the Monte Carlo simulation program MCNP6.0 [32]. The results were generated using the F8* tally, which scores all energy depositions within the sphere. A total of 48,048 MCNP simulations were performed, with a maximum relative error of 0.01. By simulating monoenergetic SAF values equal to those given in ICRP Publication 133, the sphere calculations will be provided for all 1252 radionuclides included in the decay database [22]. For ^227^Th, ^225^Ac, ^224^Ra, ^223^Ra, ^213^Bi, ^212^Bi, ^211^At, ^212^Pb, and ^149^Tb, the absorbed dose contribution from the daughter products can also be included in the absorbed dose calculations using the Bateman equations for serial decay [33]. In some cases, it is suitable to define the integration period in hours from intake to a specific time *T*_*D*_ instead of just providing the total number of disintegrations. The program gives the possibility to set a specific spherical volume in which all three decay types are interpolated separately (using the 21 simulated volumes) to estimate the absorbed dose to the volume. In the left part of Fig. 3, a spherical material of “Bone cortical (mineral)” with a density of 1.92 g/cm^3^ has been selected. The spherical volume of 10 cm^3^ has been selected with an initial activity of 1 MBq and an integration period of 1920 h. For the daughter-absorbed dose contributions, the Bateman equations have been solved using eigenvalues [34] and integrated up to a time point *T*_*D*_ (Fig. 3).

## Methods

### Software code and benchmark

The IDAC-Dose2.1 program was written in MATLAB (MathWorks, Natick, MA, USA) and compiled as a standalone program, including a graphical user interface. The SAF values generated for radionuclides with alpha, beta, and gamma decays were benchmarked against DCAL. The absorbed dose values generated by the two programs were also benchmarked for several different radiopharmaceuticals, organs, and tissues with identical results.

### Dose calculations with IDAC-Dose2.1

Absorbed doses were calculated for three radiopharmaceuticals: ^18^F-FDG, ^99m^Tc-pertechnetate, and ^131^I-iodide, which are all frequently used clinically.

#### ^18^F-FDG

Absorbed dose was calculated for ^18^F-FDG using the biokinetic model given in ICRP Publication 128 [9]. Following the EANM recommendations with a bed overlap of less than or equal to 30% and an acquisition of 3.5 min per bed position [35] for a patient weighing 73 kg, the absorbed dose was calculated for 290 MBq of administered activity.

#### ^99m^Tc-pertechnetate

The absorbed dose calculations for the orally administered activity of ^99m^Tc-pertechnetate were performed using the biokinetic model by Leggett and Giussani [36], with a slight modification assuming no uptake in the liver. The technetium model by Leggett and Giussani is not based solely on data for pertechnetate, as clinical data often show minor uptake in the liver due to the uptake of ^99^Mo-molybdate present in small amounts in the generator-produced pertechnetate. Therefore, the reported uptake in the liver is included in the organ Other, which is also in accordance with the pertechnetate model presented in ICRP Publication 53 [6].

#### ^131^I-iodide

Absorbed doses were calculated for the orally administered activity of ^131^I-iodide based on the biokinetic model presented by Leggett [37], which was later slightly modified for the family phantom series by Cristy and Eckerman [16] in ICRP Publication 128 [9]. The absorbed dose calculations for iodide were also validated against DCAL using the same input parameters.

#### IDAC spheres

Absorbed dose was calculated for a homogeneously distributed ^227^Th decay to a spherical volume with elemental compositions and densities of bone cortical (mineral) [20] with a density of 1.92 g/cm^3^ and a volume of 10 cm^3^. The absorbed dose calculation included the decay of ^227^Th and its progeny ^223^Ra, ^219^Rn, ^215^Po, ^211^Pb, ^211^Bi, and ^207^Tl for an initial activity of 1 MBq and an integration period of 1920 h.

## Results

The absorbed dose and effective dose for oral administration of ^99m^Tc-pertechnetate and ^131^I-iodide together and the results of the validation of the iodide absorbed doses with DCAL are presented in Table 1. The absorbed doses and effective dose for intravenous administration of 290 MBq ^8^F-FDG are presented in Fig. 2. The presented absorbed doses in Table 1 and Fig. 2 are given for the target organs having a tissue weighting factor in ICRP Publication 103 and for the lens of the eye. The results are identical for the two programs.

**Table 1:** Absorbed dose per unit of orally administered ^99m^Tc-pertechnetate and ^131^I-iodide activity calculated with IDAC-Dose2.1 or DCAL

Organ	IDAC-Dose2.1				DCAL	
	^99m^Tc-pertechnetate		^131^I-iodide			
	Adult male	Adult female	Adult male	Adult female	Adult male	Adult female
Adrenals	8.7E−03	1.0E−02	9.0E−02	1.0E−01	9.0E−02	1.0E−01
Brain	1.6E−03	1.9E−03	5.9E−02	8.9E−02	5.9E−02	8.9E−02
Breast	2.8E−03	2.4E−03	6.4E−02	1.4E−01	6.4E−02	1.4E−01
Colon wall	3.2E−02	3.5E−02	6.7E−02	6.4E−02	6.7E−02	6.4E−02
Endosteum (bone surface)	5.1E−03	6.8E−03	1.0E−01	1.3E−01	1.0E−01	1.3E−01
ET region	1.7E−03	2.3E−03	5.5E−01	8.2E−01	5.5E−01	8.2E−01
Eye lenses	1.0E−03	1.3E−03	6.0E−02	9.6E−02	6.0E−02	9.6E−02
Gallbladder wall	1.2E−02	8.4E−03	7.5E−02	8.9E−02	7.5E−02	8.9E−02
Heart wall	8.2E−03	7.4E−03	2.4E−01	2.5E−01	2.4E−01	2.5E−01
Kidneys	1.1E−02	1.3E−02	2.0E−01	2.3E−01	2.0E−01	2.3E−01
Liver	8.4E−03	7.8E−03	1.4E−01	1.6E−01	1.4E−01	1.6E−01
Lungs	4.7E−03	4.9E−03	2.9E−01	3.3E−01	2.9E−01	3.3E−01
Lymphatic nodes	6.7E−03	7.5E−03	9.1E−01	9.7E−01	9.1E−01	9.7E−01
Muscle	2.6E−03	3.4E−03	8.9E−02	1.3E−01	8.9E−02	1.3E−01
Oesophagus	6.6E−03	6.6E−03	2.2E+00	2.5E+00	2.2E+00	2.5E+00
Oral mucosa	2.1E−03	2.7E−03	2.2E−01	5.0E−01	2.2E−01	5.0E−01
Ovaries	–	7.4E−03	–	5.9E−02	–	5.9E−02
Pancreas	1.6E−02	1.5E−02	8.7E−02	9.8E−02	8.7E−02	9.8E−02
Prostate	3.8E−03	–	5.4E−02	–	5.4E−02	–
Red (active) bone marrow	5.2E−03	7.4E−03	2.0E−01	2.4E−01	2.0E−01	2.4E−01
Salivary glands	7.3E−03	9.2E−03	4.4E−01	6.9E−01	4.4E−01	6.9E−01
Skin	1.7E−03	2.1E−03	5.7E−02	7.2E−02	5.7E−02	7.2E−02
Small intestine wall	1.1E−02	1.4E−02	5.6E−02	6.9E−02	5.6E−02	6.9E−02
Spleen	9.2E−03	1.3E−02	1.0E−01	1.2E−01	1.0E−01	1.2E−01
Stomach wall	3.1E−02	3.1E−02	5.8E−01	5.9E−01	5.8E−01	5.9E−01
Testes	1.9E−03	–	2.1E−02	–	2.1E−02	–
Thymus	3.0E−03	3.1E−03	2.4E+00	2.1E+00	2.4E+00	2.1E+00
Thyroid	4.2E−02	5.1E−02	3.6E+02	4.4E+02	3.6E+02	4.4E+02
Urinary bladder wall	6.0E−03	8.0E−03	1.2E−01	1.3E−01	1.2E−01	1.3E−01
Uterus/cervix	–	7.3E−03	–	6.8E−02	–	6.8E−02
Effective dose (publ. 60) [mSv/MBq]	1.3E−02	1.5E−02	1.9E+01	2.2E+01		
Effective dose (publ. 103) [mSv/MBq]	1.4E−02		1.6E+01		1.6E+01	

Data are given in mGy/MBq unless otherwise noted. Effective dose was calculated using the two sets of tissue weighting factors given in ICRP Publications 60 and 103

For intravenous administration of ^18^F-FDG, the highest absorbed doses are received by the urinary bladder wall and heart wall for both the male and female phantoms. The effective dose was calculated using the tissue weighting factors from ICRP Publication 103 as 0.016 mSv/MBq (4.7 mSv for an administration of 290 MBq). For oral administration of ^99m^Tc-pertechnetate, the highest absorbed dose was received by the thyroid for both the male and female phantoms. The effective dose calculated using the tissue weighting factors of ICRP Publication 103 was 0.014 mSv/MBq. For oral administration with medium thyroid uptake of ^131^I-iodide, the highest absorbed dose for both phantoms was received by the thyroid. The effective dose calculated using the tissue weighting factors from ICRP Publication 103 was 16 mSv/MBq.

The absorbed dose estimated for homogeneously distributed ^227^Th decay at a time interval of 80 days (*T*_*D*_ = 1920 h) and an initial activity of 1 MBq to a sphere of 15 cm^3^ consisting of material type “Bone cortical (mineral)” with a density of 1.92 g/cm^3^ was 110 and 563 Gy when including the decay of the daughter products ^223^Ra, ^219^Rn, ^215^Po, ^211^Pb, ^211^Bi, ^207^Tl, and ^211^Po of ^227^Th into the calculation.

## Discussion

Absorbed doses have been calculated for three different clinical examinations with radiopharmaceuticals using IDCA-Dose2.1. The software offers the option of calculating the absorbed dose to 47 different organs and tissues. Here, the absorbed doses are presented for organs and tissues given a tissue weighting factor in ICRP Publication 103 [19] and the absorbed dose to the lens of the eye. The tissue weighting factor is defined to reflect the relative contribution of the organ and tissue to the detriment for stochastic effects.

The effective dose of 4.73 mSv was calculated for a 292-MBq intravenous administration of ^18^F-FDG, which corresponds to an effective dose coefficient of 0.016 mSv/MBq. The same biokinetic model for intravenous administration of ^18^F-FDG was used in ICRP Publication 128 [9] with an effective dose coefficient of 0.019 mSv/MBq. The absorbed doses for ^99m^Tc-pertechnetate in ICRP Publication 128 [9] are based on the biokinetic model presented in ICRP Publication 53 [6] and the SAF values on the previous stylised adult phantoms. For orally administered ^99m^Tc-pertechnetate, an effective dose coefficient of 0.016 mSv/MBq was calculated using the sets of tissue weighting factors given in ICRP Publication 60 [15]. In this paper, the effective dose coefficient for the same procedure was estimated to be 0.014 mSv/MBq when using an updated biokinetic model, revised phantoms, updated tissue weighting factors, updated nuclear decay data, and a new computational framework. For oral administration with medium thyroid uptake of ^131^I-iodide, the effective dose in ICRP Publication 128 [9] was estimated to be 22 mSv/MBq and, in this paper, using the same biokinetic model, the coefficient was estimated to be 16 mSv/MBq, which corresponds to a 28% reduction. In general, is it not possible to determine explicitly which factor contributes most to the change in the coefficient. However, in the case for ^131^I-iodide where 96.4% of effective dose value arises from irradiation of the thyroid is the dominating factor for the effective dose reduction in the change of the tissue weighting factor for the thyroid from 0.05 in ICRP Publication 60 to 0.04 in ICRP Publication 103. The new calculation methods from more realistic anatomical and biokinetic models will provide more accurate absorbed dose results and probably allow more accurate estimations of the radiation-induced risk of stochastic effects to patients examined by nuclear medicine.

Before the official SAF values of the ICRP/ICRU reference phantom given in ICRP Publication 133 [21] were published, absorbed dose estimations were based directly on Monte Carlo simulations using the adult reference computational phantoms presented in ICRP Publication 110 [20]. In three previously published papers, the effective dose coefficient for ^18^F-FDG has been estimated to be 0.018 mSv/MBq [38], 0.017 mSv/MBq [24], and 0.019 mSv/MBq [39, 40], respectively, which is in satisfactory agreement with the 0.016 mSv/MBq.

All absorbed doses presented in this paper are higher for the female phantom except in the case of the liver for ^99m^Tc-pertechnetate and thymus for ^131^I. The reason for this could be that, except for the sex-specific modelling of the alimentary tract, the pharmacokinetics of the radiopharmaceutical is not gender-specific. If the highest absorbed dose contribution arises from non-penetrating radiation, the higher absorbed doses to the Reference Female reflect the smaller masses of the female phantom than the male phantom. To consider the differences in mass, sex-specific biokinetic models need to be created.

The introduction of the new source region Blood allows the possibility of including compartment modelling in the program. To apply the source region Blood, a separation of the circulating blood from the specific source region is necessary. This is not always possible when the measured data points are generated from images of medical devices. Therefore, this source region should only be used on compartmental models in which the circulating blood is defined as a separate compartment and carefully applied in descriptive modelling, because the blood acts only as a transfer compartment in descriptive modelling and is distributed to other source regions and no separation between the tissue and the blood is needed.

A separate module for spherical structures and the possibility of adjusting for different activity distributions within an organ or tissue has also been included in the program to facilitate finding solutions to other clinically relevant problems. The possibility of adjusting the integration period in the calculation in the sphere module is intended to remove the problem for daughter products with long half-lives, such as ^221^At, which has a half-life of 7.21 h, and a daughter product of ^207^Bi, which has a half-life of 32.9 years, and for which integrating to infinity would probably not be realistic. The absorbed dose from ^221^At to the cortical bone (1 MBq, 80 days, without contribution from decay products) was estimated to be 110 Gy. The same simulation for a water sphere of density 1 g/cm^3^resulted in an absorbed dose of 211 Gy. The ratio in absorbed doses between the cortical bone and the water is 0.521 (211 Gy/110 Gy) which is also the ratio in densities between the two materials (1.92 g cm^−3^/1 g cm^−3^ = 0.521). This is because most of the emitted radiation is locally absorbed. However, for ^18^F which has a high fraction of photon emission this ratio is 0.57 (23 Gy/41 Gy).

Dose estimations are an important part of quality assurance programs in medical radiology. Very few validated and freely available dose calculation programs are currently available for nuclear medicine. IDAC-Dose2.1 provides new users the opportunity to perform absorbed dose estimates based on their own biokinetic data. For users who have created in-house programs or have access to commercially available programs, IDAC-Dose2.1 can serve as an important tool for inter-comparisons and quality assurance. A weakness of using reference phantoms is the lack of applicability to generate accurate patient-specific dosimetry. Currently, the ICRP has only published SAF values for the adult phantoms [21], but is now developing updated SAF values for pre-adults; when these are available, they will also be included in IDAC-Dose2.1. Until the ICRP publishes the SAF values for pre-adults, an automatic function in the program (IDAC-Dose2.1) facilitates absorbed dose calculations with IDAC-Dose1.0 [2, 3] to the stylised pre-adult phantoms using recalculations of the cumulated activity per administered activity for the adult male.

## Conclusions

Calculations with IDAC-Dose2.1 using the ICRP adult reference computational phantoms will lead to improved absorbed dose calculations compared to previous programs based on stylised mathematical models. IDAC-Dose2.1 was created to implement clinically the most recent ICRP computational framework for internal dose assessment given in ICRP Publication 133 and is suitable for patients in nuclear medicine. This computational framework is not always consistent with the MIRD schema (e.g., regarding the definition of the source regions Other and Blood).

Absorbed dose and effective dose calculations were performed using the latest biokinetic models for ^18^F-FDG, ^99m^Tc-pertechnetate, and ^131^I-iodide. In this way, IDAC-Dose2.1 was validated against DCAL with identical results. The two ICRP programs have been harmonised and based their calculations on the same computational framework, so that identical radiation exposures give the same absorbed dose independent of the situation for which they are estimated.

The IDAC-Dose2.1 program is free software for research and available for download at www.idac-dose.org.

## Abbreviations

DCAL: Dose and Risk Calculation
FDG: Fluorodeoxyglucose
ICRP: International Commission on Radiological Protection
IDAC: Internal Dose Assessed by Computer
SAF: Specific absorbed fraction
